# Testosterone administration does not alter the brain activity supporting cognitive and affective empathy

**DOI:** 10.1016/j.cpnec.2022.100134

**Published:** 2022-04-02

**Authors:** Andrei Alexandru Puiu, Mikhail Votinov, Ute Habel, Kerstin Konrad

**Affiliations:** a Department of Psychiatry, Psychotherapy and Psychosomatics, Faculty of Medicine, RWTH Aachen University, Aachen, Germany; b Child Neuropsychology Section, Department of Child and Adolescent Psychiatry, Psychosomatics and Psychotherapy, Faculty of Medicine, RWTH Aachen University, Aachen, Germany; c Institute of Neuroscience and Medicine, JARA-Institute Brain Structure Function Relationship (INM 10), Research Center Jülich, Jülich, Germany; d JARA-Brain Institute II, Molecular Neuroscience and Neuroimaging, Research Center Jülich, Jülich, Germany

**Keywords:** Testosterone administration, fMRI, Affective empathy, Cognitive empathy, Placebo-controlled

## Abstract

Although there is evidence that testosterone has deteriorating effects on cognitive and affective empathy, whether testosterone administration influences both routes to understanding others has not yet been simultaneously investigated.

We conducted a functional magnetic resonance imaging (fMRI) pharmacological study using a within-subjects, randomized, placebo-controlled, double-blind crossover design to examine the effects of 100 mg transdermal testosterone administration on brain activation during a task that examines affective and cognitive empathy simultaneously in a sample of 23 healthy right-handed adult men.

Relative to placebo, testosterone did not alter affective or cognitive empathy functional brain networks. Instead, the task yielded activation in the canonical networks associated with both types of empathy. Affective empathy yielded activation in the inferior and middle frontal gyri, inferior temporal gyri, and the cingulate cortex. Cognitive empathy was associated with activation of the temporoparietal junction, medial prefrontal cortex, middle and inferior temporal gyri, and temporal pole. Behaviourally, testosterone administration decreased error rates and increased participants’ confidence in their responses regardless of response accuracy. Independent of testosterone administration, participants reported higher affective responses during emotionally negative scenarios.

Even though our results provide further evidence that testosterone administration in healthy men does not alter brain activity underlying cognitive and affective empathy, testosterone administration does influence the empathic concern and hence socio-cognitive processes. The reproducibility and variability of the current and previous findings should nevertheless be addressed in upcoming studies.

## Highlights

• High testosterone concentrations are associated with deficient social cognition such as higher aggression and lower empathy.
• We investigated whether 100mg testosterone administration alters the brain activity supporting empathy in 23 healthy men.
• Results showed that neither affective nor cognitive empathy brain network activity was altered by testosterone.
• Independent of testosterone, participants responded with more affective empathy and concern during negative scenarios.
• Our results show that acute testosterone administration in healthy men does not alter most socio-cognitive processes.

## 1 Introduction

Empathy or the ability to understand others, identify and tune in with their affective states is fundamental for social functioning and prosocial behaviour [1]. Empathy is an umbrella term that encompasses different behaviours and mental states supported by cognitive and affective processes. Cognitive empathy is a deliberate and effortful process aimed at internalizing someone's perspective by attributing their beliefs, intentions, or desires (otherwise called mental states or theory of mind; ToM) to someone else [1,2]. It is therefore the ability to understand and recognize others' mental states including their emotions, goals, or beliefs from limited observational information [31,52]. Affective empathy, on the other hand, entails the vicarious and simultaneous feeling of and responding to others' emotions without any direct emotional stimulation to oneself [12,18]. This is thus a dynamic process extending from simple forms of emotion contagion to rather multi-level forms of cognitive perspective-taking [19]. Nonetheless, the strict dichotomization of cognitive and affective empathy as per these definitions might not hold up under close scrutiny since empathy is a flexible and adaptive phenomenon that is affected by many factors (for a review, see [18].

Previous studies showed that these two forms of empathy rely on different brain networks and follow different developmental pathways, with cognitive empathy developing ontogenetically later than affective empathy [31,49]. Pooled meta-analyses on studies investigating cognitive empathy (or theory of mind) revealed a core network comprising the medial prefrontal cortex, temporoparietal junction, posterior cingulate, precuneus, and the orbitofrontal cortex [13,47]. Affective empathy has been typically investigated with paradigms probing empathy for pain. Core brain regions associated with someone else's suffering are the bilateral insula and anterior and middle cingulate cortices [35]. Additional areas recruited during the (perceived) experience of pain extend to the somatosensory, inferior parietal, ventromedial, and superior temporal cortices. Complementary to affective empathy, witnessing someone else's suffering often elicits empathic concern or the feeling of care that is paired with an intrinsic wish to alleviate others' suffering [50]. Unlike affective empathy which elicits a reaction to negative affect, empathic concern is associated with positive affect and reflects how one feels towards another and often evokes activity in the ventral striatum [34].

Previous studies have investigated cognitive and affective components of empathy in isolation [13,20] or by directly comparing them [9,31,57]. Kanske and colleagues (2015) employed a task that simultaneously measured cognitive and affective empathy and their results highlighted separable neural networks for the two routes of understanding others including the anterior insula for the affective and the ventral temporoparietal junction for cognitive empathy. Neural activity in these areas also predicted the respective behavioural responding. In other words, strong negative emotions limited one's ability to reflect on another's perspective and increased self-reported negative affect [33]. Similarly, [48] found that empathy is a multi-dimensional hierarchical model supported predominantly by cognitive processes (engaged when mentalizing is required), some affective processes (engaged in managing and responding with emotions), and combined processes that engage both cognitive and affective mechanisms. Therefore, despite relying on somewhat segregated neural systems, the networks supporting cognitive and affective empathy are interconnected and act synergistically.

Several streams of evidence showed an effect of sex steroids and in particular of testosterone on socio-cognitive processes. Testosterone is an important sex hormone in the human endocrine system which affects physiology and behaviours. Changes in mental states and behaviour may be explained by differential binding of endogenous (changing concentrations) as well as exogenous sex steroids (due to different binding properties) to receptor sites of brain regions involved in socio-emotional processing (frontal cortex and limbic areas) [10,63]. But individual difference in the circulating and pharmacologically-manipulated levels of testosterone are often weak predictors of individual differences in social behaviours [3,6,26]. Previous studies linked testosterone with competitive, aggressive, strategic, and dominance behaviours. A commonality across the heterogeneous phenotypic effects associated with testosterone is a pronounced non-social cognition and blunting of mentalistic social information processing. Testosterone administration, for instance, has been associated with a reduced ability to recognize and ascertain emotions (i.e., from the eyes or mouth region of faces [56]) or with heightened self-confidence levels [17], with effects being (weakly) mediated by prenatal testosterone exposure. Similarly, testosterone administration increased social discounting [[25], [64]] and reduced affect and paternal care [27,44]. Some behavioural studies showed an effect of testosterone on diminishing cognitive empathy [14,40,56]. In women, testosterone has been negatively associated with empathic processes such as perspective-taking [39] and complex emotion recognition [8,56]. But others found no evidence of these effects [[14], [32], [38]]. Several other studies, however, established a link between empathy measures and aggression showing that high empathy is linked to diminished aggressive behaviours [5,22]. A similar relationship has also been observed between empathy and testosterone levels, with high empathy being associated with low aggression at low or moderate testosterone levels [4,37]. At neural level, a detrimental effect of testosterone has been observed on the functional connectivity between areas involved in social information processing, the amygdala, and the orbitofrontal cortex [53,58]. These findings show that testosterone can influence social decision-making [7,21] either through top-down mechanisms that alter cognitions about other people or via bottom-up processes altering prepotent affective responses [15,21]. Rather than working independently, however, cognitive and emotional processes influence and interact with each other [16,42,55]. To what extent testosterone could alter the neural responses to cognitive and affective empathy simultaneously, however, has not yet been investigated.

Pharmacological experiments that manipulate testosterone concentrations are essential in investigating testosterone's causal effects. Therefore, the main aim of this study was to systematically investigate the effects of transdermal testosterone administration on the neural networks associated with cognitive and affective empathy. For this purpose, we utilized an empathy task developed by Kanske and colleagues (2015) that simultaneously manipulates cognitive and affective empathy. The paradigm employed naturalistic videos depicting emotionally negative or neutral video scenarios giving rise to factual reasoning (noToM requirements) or to theory of mind questions (ToM). Despite the distinction between the two types of empathy outlined above, if testosterone modulates empathy, we expect it will act similarly on the neural systems known to mediate the two processes. Additionally, we were interested in whether, independent of testosterone manipulation, the task disentangles the two canonical networks involved in cognitive and affective empathy.

Therefore, independent of testosterone manipulation, we hypothesized that:

1. The paradigm will elicit dissociable functional activity in the canonical brain networks associated with cognitive and affective empathy.
2. Participants' subjective affective responses (how they felt themselves) assessed using valence ratings ranging from positive to negative will be more negative after emotionally-negative scenarios compared to emotionally neutral scenarios.

Regarding the modulatory effects of testosterone, findings suggest a dampening of empathic abilities following testosterone administration. Thus, we hypothesized that:

3. Relative to placebo administration, testosterone will reduce affective and cognitive empathy abilities alike evidenced by decreasing the functional activity of the canonical brain networks associated with affective and cognitive empathy.
4. Increased testosterone levels will be associated with reduced negative affect (self-reported affect valence) and empathic concern. This is consistent with the hypothesis that supraphysiologic testosterone dampens cognitive and affective abilities.

As the paradigm also investigates confidence in own behavioural responding, we exploratorily examined the effect of testosterone on self-reported response confidence.

## 2 Materials and methods

### 2.1 Sample

The current paper is part of a larger neuroendocrine project aimed at investigating the effects of modulatory effects of administered testosterone on empathy, risk-taking, and loss aversion (see [59] for a preprint). Power calculations have been performed and a sensitivity curve depicting the power of the current design and sample to detect effects is available in the supplement. Specifically for this study, anticipating a moderate effect size (ʃ^2^: 0.15) using two predictors (empathy network activation and supraphysiologic testosterone concentrations), α = 0.5, β = 0.8, the minimum required sample size was 43 participants. We included a 10–15% drop-out rate in the original calculation. However, the approach to ignore the 10–15% of a sample due to attrition is incorrect and the current sample is likely underpowered for the initially specified effect. Twenty-eight healthy young right-handed males were recruited. Approvals were granted by the local ethical committee of the Medical Faculty of the RWTH Aachen University and the study was performed according to the declaration of Helsinki. We conducted the experiment at the RWTH Aachen University Hospital between September 2018 and January 2019. Participants were recruited from the general population of Aachen (Germany) and surrounding areas by means of public advertisements. Participants were naïve to prior testosterone administration upon participation in this fully-randomized, within-subject, placebo-controlled study. They were informed that testosterone and placebo will be administered at random in two separate sessions. All participants provided written informed consent and were screened for psychiatric conditions. Additional exclusion criteria were current or past use of psychotropic medication, endocrine or cardiac disorders, left-handedness, habitual smoking, hearing and visual deficits, history of psychiatric disorders or neurological insult(s), and irregular sleep patterns. After recruitment, two participants dropped out, one participant did not comply with the task instructions, and two additional participants did not complete both testing sessions. In total, data from 23 participants (23.8 ± 3.22 years; BMI: 23.7 ± 2.23) entered the analyses. Participants received financial compensation upon study completion. Participants were scanned at the same time of day on two separate days with a washout interval of at least one week. When compared to normative data from the general population, participants in the current study did not differentiate on self-reported empathy (using the short version of empathizing-systemizing self-assessment scales [46]). The sample had mean empathizing values of 12.6 (±3.71) and 13.3 (±3.71) following placebo and testosterone administration, respectively (compared to an average of 13.79 ± 5.88) [46].

### 2.2 Testosterone administration

Treatment administration was fully-randomized according to a crossover, repeated-measures, placebo-controlled design. Participants received one 4-g tube containing 100-mg testosterone (Testotop®, Galenpharma GmbH, Wittland, Kiel, Germany) and another tube containing 100-mg placebo gel across two separate sessions. The testosterone sample contained additional ethanol (96%) that acted as a testosterone solvent to aid transdermal penetration, polyacrylate (carbomer 980), propylene glycol for viscosity control, trometamol, disodium EDTA, and purified water. This is a well-established single-dose testosterone administration procedure that was previously validated [43]. Physiological effects of the testosterone administration were observed in serum concentrations which were significantly increased relative to baseline and placebo administration 1.5 h after testosterone administration. Several other studies using similar administration protocols and various behavioural paradigms have reported effects after a minimum 1-h delay post-administration [14,15]. For the chosen dosage, no side effects have been reported in this or previous studies to date. We performed the fMRI measurement 1.5 h after treatment administration.

### 2.3 Procedure

Participants were invited to the laboratory twice within an interval of a minimum of one week between sessions. Before the testing days, participants were reminded to fast overnight and not to eat for 2 h before testing. Both testing days followed the same procedure. After arrival at the laboratory between 8.30 a.m. and 8.45 a.m., participants were briefed about the procedure and signed the consent form. A first blood sample (10 mL) was collected around 9 a.m. serving as a baseline measurement. After collecting the first blood sample, the transdermal gel was applied by the same research assistant to participants' scapular area. The gel was allowed 15min to dry out and be absorbed into the bloodstream following which participants were allowed to dress. After administration, participants were kept under observation in the laboratory and were instructed to refrain from physically and psychologically intensive tasks. One-and-a-half hours after the application of the transdermal treatment, participants were taken to the MR scanner. A second blood sample was taken before the MR session started (T_1_). Before the MRI measurements, participants were screened for MR counterindications and were then instructed to position themselves on the scanner bed as comfortably as possible and to try to relax. Head movement was minimized by foam pads that were placed between the RF-coil and the participant's head. Further instructions during the scan session were given by the intercom. The last blood sample was taken after the MR session (T_2_). In total, three blood samples were collected. Each session concluded with a debrief and an evaluation where participants were asked to indicate whether they thought they received testosterone or placebo on the corresponding testing day. After the second session, participants were debriefed, given payment, and asked to indicate the day of administration to control for blindness regarding treatment administration.

### 2.4 Cognitive and affective empathy task

**Fig. 1:**
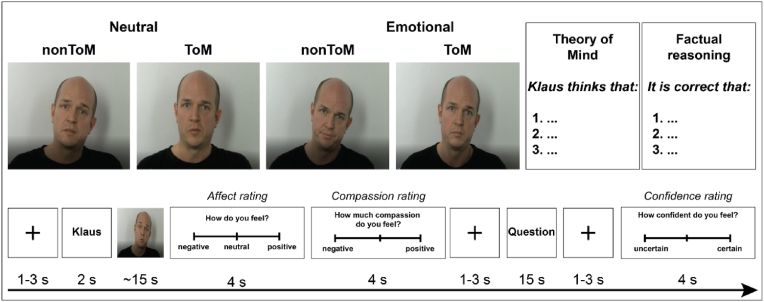
EmpaToM schematic trial sequence. Four different videos were presented to participants according to a 2 (video emotionality: neutral vs emotionally negative) x 2 (ToM requirement: noToM vs ToM) design.

To simultaneously investigate cognitive and affective empathy, we used an adapted version of the EmpaToM [31]; Fig. 1). The task presents participants with a sequence of stimuli per trial. Following a fixation cross (1–3s), the participants see a name (1s) associated with the person who will speak in a subsequent short video (roughly 15s). The videos differ in emotional valence in that some videos recount emotionally neutral while others recount emotionally negative content. The videos also differ in the questions they give rise to (non-theory of mind vs. theory of mind questions; noToM vs ToM). Following each video, participants rated how they felt (4s; (“How do you feel”; “very negative” to “very positive”) and how much compassion they felt (a marker of empathic concern) for the person in the video they just saw (“How much compassion do you feel”; “none” to “very much”). Participants responded by moving a slider using the index and middle finger of the right hand. After a fixation cross (1–3s), participants are presented with a multiple-choice question with three response options. The questions require either a theory of mind (ToM; “Klaus thinks that …”) or factual reasoning inference (noToM; “Is it correct that …”) on the content of the previous video. Participants had 14s to respond to the question. After a fixation cross (1–3s), a confidence rating was presented asking the participant to indicate how confident they were about their chosen answer (4s). Twelve trials per condition were presented and each actor recounted one story per condition. In total, 12 different actors (six male) recounted the scripted stories. Overall, the task comprises 48 videos and has been designed with the following semantic characteristics consistent: number of words, number of characters, number of predicates, changes in tense, and complexity of the sentences (a detailed description of task validation and example stories and questions per each condition, see Ref. [31] and supplement).

### 2.5 Questionnaire selection

We assessed trait impulsivity with the Baratt Impulsiveness Scale (BIS-11 [41]), trait aggression with the Buss and Perry Aggression Questionnaire (BPAQ; [11], and empathizing and systemizing with the short version of the empathizing and systemizing self-assessment scales [46].

### 2.6 Hormonal profiles

Testosterone serum concentrations were analysed by electrochemiluminescence immunoassays (ECLIA, Roche®Diagnostics GmbH) under strict internal and external quality control at the Clinical Chemistry, Haematology, Virology, and Microbiology Laboratory Diagnostic Centre (LDZ) of the RWTH Aachen University Hospital. The inter-assay coefficient for testosterone was 2.4% with a lower detection limit of 0.09 nmol/L. The intra-assay coefficient was below 3%.

### 2.7 MRI data acquisition

Imaging data were acquired using a Siemens 3 T Prisma scanner (Siemens AG; Erlangen, Germany) equipped with a 12-channel head matrix coil located at the Department of Psychiatry, Psychotherapy and Psychosomatics, RWTH Aachen University. Functional scans were acquired using a T2*-weighted echo-planar imaging (EPI) sequence with a slice thickness = 3 mm, TR = 2000 ms, TE = 28 ms, flip angle = 77, interleaved ascending. Structural scans were acquired using a T1-weighted magnetization prepared rapid gradient echo (MPRAGE) sequence with the following acquisition parameters: TR = 2300, TE = 2.98 ms, flip angle = 9°, FOV = 256 × 256 mm, 176 slices, voxel size = 1 mm³, interleaved, distance factor: 50%, GRAPPA accel. factor PE = 2.

### 2.8 Treatment manipulation check and behavioural data analyses

We assessed whether treatment manipulation was successful using a 2 (treatment: placebo vs testosterone) x 3 (time: baseline, T1, T2) repeated measures MANOVA design. In the EmpaToM paradigm, subjective valence ratings (affect and concern), as well as task performance (reaction times and error rates) were analysed using a 2 × 2 x 2 full-factorial repeated-measures MANOVA with treatment (testosterone vs placebo), video emotionality (negative vs neutral), and ToM requirement (noToM vs ToM) as within-subject factors. We deviated from the protocol proposed by Kanske et al. [31] in terms of analysing the RT and accuracy data because combining RTs and accuracy rates into a z-transformed aggregate score makes our effects difficult to interpret. Moreover, since the aim of this paper is to investigate the effects of testosterone on empathy, RTs are redundant given the paradigm is not a reaction time task like the SSRT, for instance. Here, the RT is basically a time interval where the participant must make a decision. Participants could be very fast in responding albeit wrong. Nevertheless, we analysed and reported the RT results to be consistent with previous literature. Participants’ subjective affective response (how they felt themselves) and empathic concern (how they felt towards the other) were assessed with valence ratings ranging from positive to negative. Behavioural empathy was assessed with the valence ratings (emotionally neutral vs negative).

### 2.9 fMRI data analyses

Images were analysed using SPM12 (http://www.fil.ion.ucl.ac.uk/spm/) running under MATLAB 2019b (The Math-Works, Natick, MA). We realigned the time series according to a two-pass procedure using the first image (first pass) and the mean image (second pass) as reference. All volumes were coregistered to their mean EPI and subsequently used to determine spatial normalization parameters using the unified segmentation approach. The transformation matrix obtained by normalizing the anatomical image was then used to transform the time series into the standard Montreal Neurological Institute (MNI) space. During normalization, all images were resampled to a voxel size of 2 × 2 × 2 mm³. The normalized images were spatially smoothed using an isotropic Gaussian kernel of 8 mm full-width-at-half-maximum. A high-pass temporal filter at 128s was applied to remove low-frequency drifts. After image preprocessing, we carried out the statistical analyses using the general linear model. We modelled the onset and duration of the four video types, their corresponding questions, and the rating epochs. We then convolved these regressors with a canonical hemodynamic response function (HRF). Effects of head motion were accounted for and entered the design matrix as effects of no interest. We calculated the contrast images for the affective (emotionally negative > emotionally neutral videos) and cognitive empathy (noToM > ToM) contrasts by applying linear weights to the parameter estimates. The estimates entered into a one-sample *t*-test for random-effects analysis. Second-level random effects modelling tested the null hypothesis of zero difference across participants between the testosterone and placebo conditions. Whole-brain analyses were run using FWE-corrected voxel-level significance, thresholded at *p* < .05 throughout. A full factorial 2 × 2 × 2 MANOVA (testosterone/placebo, negative/neutral emotionality, ToM/noToM) was modelled using event/related onset times of the task stimuli and tested for whole-brain effects. Given that the chosen statistical thresholding can be considered rather conservative concerning the subtle effects of endocrine manipulation, we ran exploratory analyses at voxel-level FWE-corrected with an α < 0.01. Results are visualized by superimposing the statistical parametric maps onto a high-resolution canonical T1-image.

## 3 Results

### 3.1 Manipulation checks

#### 3.1.1 Testosterone concentrations

**Fig. 2:**
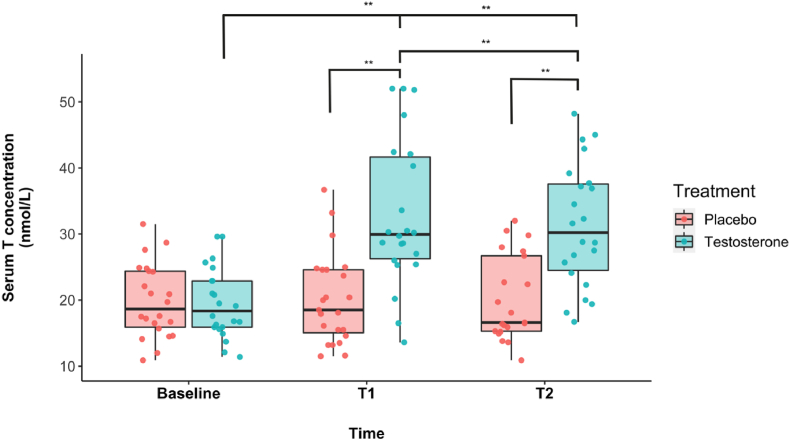
**Serum testosterone concentration.** 100-mg transdermal testosterone administration increased total serum T levels significantly relative to placebo at T1 and remained consistently significantly elevated at T2. Jittered dots represent individual data points.

The RMANOVA revealed a significant main effect of treatment (F_1,19_ = 31.9, p < .001, η^2^_p_ = .63) and time (F_2,38_ = 24, p < .001, η^2^_p_ = .56). The interaction between time and treatment was also significant, F_2,38_ = 26.5, p < .001, η^2^_p_ = 58 (Fig. 2). The results show that testosterone levels changed differentially across time with transdermal testosterone relative to placebo administration. Post-hoc analyses showed a mean difference of 8.18 nmol/L between placebo and testosterone administration, *t*(1.5) = 5.65, *p*_*Bonf*_ < .001, Cohen's d = 1.26. Testosterone levels were 7.07 nmol/L higher at T_1_ relative to baseline, *t*(1.3) = 5.59, *p*_*Bonf*_ < .001, Cohen's d = 1.25 and 6.16 nmol/L higher at T_2_ relative to baseline, *t*(0.9) = 6.49, *p*_*Bonf*_ < .001, Cohen's d = 1.45. Testosterone levels did not differ significantly between T_1_ and T_2_. Means (and SDs) are available in Table 1. Cortisol concentrations are available in the supplement.

**Table 1:** Time x Treatment means and standard deviations.

Treatment	Time	M	SD
Placebo	T0	19.76	5.77
	T1	19.76	6.52
	T2	20.38	6.50
Testosterone	T0	19.54	5.64
	T1	33.69	11.32
	T2	31.23	9.75

### 3.2 Behavioural results

#### 3.2.1 Task performance

There were no significant main effects for reaction times (treatment: F_1,22_ = 1.15, *p* = .29; video emotionality: F_1,22_ = 0.36, *p* = .55; ToM: F_1,22_ = 1.34, *p* = .25). There were also no significant interaction effects for video emotionality and treatment (F_1,22_ = 3.73, *p* = .06) and for ToM requirement and treatment (F_1,22_ = 0.01, *p* = .9). Only the interaction between video emotionality and ToM requirement reached significance (F_1,22_ = 5,78, *p* = .02). The three-way interaction of video emotionality x ToM requirement x treatment was also not significant (F_1,22_ = 1.11, *p* = 0.3).

The analysis of error rates showed a significant main effect of treatment (F_1,22_ = 6.24, *p* = .02, η^2^_p_ i= .22) and ToM requirement (F_1,22_ = 15.38, *p* < .001, η^2^_p_ = .41) but no significant effect for video emotionality (F1,22 = 0.02, *p* = .87). There were no significant interaction effects for video emotionality and treatment (F_1,22_ = 0.53, *p* = .47), for ToM requirement and treatment (F_1,22_ = 2.68, *p* = .11) and for video emotionality and ToM requirement (F_1,22_ = 0.09, p = .75) The three-way interaction of video emotionality by ToM requirement by treatment (F_1,22_ = 0.002), *p* = .96 was not significant. Error rates were higher following placebo (42.7% ± 14.8) compared to testosterone (37.6% ± 17.1) administration, *t* = 2.49, *p* = .02. Error rates were also higher during noToM (44.3% ± 16.4) relative to ToM conditions (36.1% ± 15.6), *t* = 2.49, *p*_*Bonf*_ = .02. None of the correlations between task performance and questionnaires were significant (correlation matrices are available in the supplement).

#### 3.2.2 Affect ratings

**Fig. 3:**
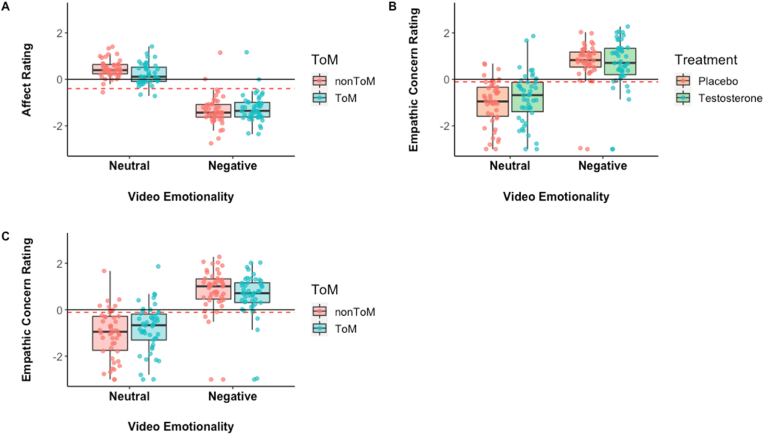
Subjective valence ratings. (A) Affect valence ratings as a function of theory of mind requirement; (B) empathic concern valence ratings as a function of treatment administration; (C) empathic concern valence ratings as a function of theory of mind requirement. Dotted red lines indicate the observed grand mean. Whiskers show 95% confidence intervals. Jittered dots represent individual data points. (For interpretation of the references to colour in this figure legend, the reader is referred to the Web version of this article.)

There was no effect of treatment administration on the affect ratings (F_1,22_ = 0.5, *p* = .45). The RMANOVA analysis revealed a significant main effect of video emotionality, F_1,22_ = 147.5, *p* < .001, η^2^_p_ = .79. The interaction between video emotionality and ToM requirement was also significant, F_1,22_ = 18.7, *p* < .001, η^2^_p_ = .45 (Fig. 3A). Post-hoc tests showed that participants rated emotionally negative videos more negatively (−1.29 ± 0.61) than the emotionally neutral videos (0.33 ± 0.3), t(0.13) = 12.14, *p* < .001. Participants rated the emotional ToM more negative than the neutral ToM videos (t = 10.51, *p*_*Bonf*_ < .001, Cohen's d = 2.19) and the neutral noToM videos (t = 12.14, *p*_*Bonf*_ < .001, Cohen's d = 2.53). Negative noToM videos elicited more negative affect than neutral ToM videos (t = 11.18, *p*_*Bonf*_ < .001, Cohen's d = 2.33) and than neutral noToM videos (t = 12.86, *p*_*Bonf*_ < .001, Cohen's d = 2.68).

#### 3.2.3 Empathic concern

There was no effect of treatment administration on the empathic concern ratings (F1,22 = 0.47, *p* = .49). The analysis revealed a significant main effect of video emotionality, F_1,22_ = 88.8, *p* < .001, η^2^_p_ = .79. The interactions between video emotionality and treatment (F_1,22_ = 4.37, *p* = .048, η^2^_p_ = .16) and between video emotionality and ToM requirement (F_1,22_ = 19.6, *p* < .001, η^2^_p_ = .47) were significant. Post-hoc tests showed that participants reported more concern during the negative compared to neutral videos, t = 9.31, *p* < .001. On placebo administration, participants showed more concern during emotionally-negative relative to emotionally neutral videos, t = 9.47, *p*_*Bonf*_ < .001, Cohen's d = 1.97. Participants also reported more concern during emotionally-negative relative emotionally-neutral videos following testosterone administration, t = 8.04, *p*_*Bonf*_ < .001, Cohen's d = 1.67. Relative to placebo, testosterone administration decreased participants' concern ratings for emotionally-neutral videos more than for emotionally-negative videos, t = 7.36, *p*_*Bonf*_ < .001, Cohen's d = 1.53 (Fig. 3B). Similarly, participants' concern ratings were lower for emotionally negative videos after testosterone administration than for emotionally-neutral videos following placebo administration, t = 8.13, *p*_*Bonf*_ < .001, Cohen's d = 1.69. Participants showed more concern for the emotional ToM relative to the neutral ToM (t = 7.86, *p*_*Bonf*_ < .001, Cohen's d = 1.69) and the neutral noToM videos (t = 9.15, *p*_*Bonf*_ < .001, Cohen's d = 1.9). Negative noToM videos elicited more concern than neutral ToM (t = 8.97, *p*_*Bonf*_ < .001, Cohen's d = 1.87; Fig. 3C) and than neutral ToM (t = 8.97, *p*_*Bonf*_ < .001, Cohen's d = 1.87) and neutral noToM videos.

#### 3.2.4 Exploratory analysis of self-confidence ratings

The analysis yielded a significant main effect of treatment (F_1,22_ = 14.98, *p* < .001, η^2^_p_ = .41) and ToM requirement (F_1,22_ = 5.2, *p* = .03, η^2^_p_ = .19). Participants were more confident in the correctness of their answers following testosterone compared to placebo administration (M_testosterone_ = 1.4 ± 0.8, M_placebo_ = 1.1 ± 0.9; t = 3.87, *p*_*Bonf*_ < .001) regardless of response accuracy. Participants were also more confident in their answers during videos requiring ToM inferences relative to the noToM videos (M_ToM_ = 1.08 ± 0.7, M_noToM_ = 0.92 ± 0.7; t = 3.87, *p*_*Bonf*_ < .001 Cohen's d = 0.81).

### 3.3 fMRI results

#### 3.3.1 Testosterone treatment

There was no effect of transdermal testosterone treatment with the conventional FWE-corrected voxel-level significance thresholded at *p* < .05 nor did the exploratory analyses (thresholded at voxel-level FWE-corrected with α < 0.01) reach significance.

#### 3.3.2 Affective empathy

**Fig. 4:**
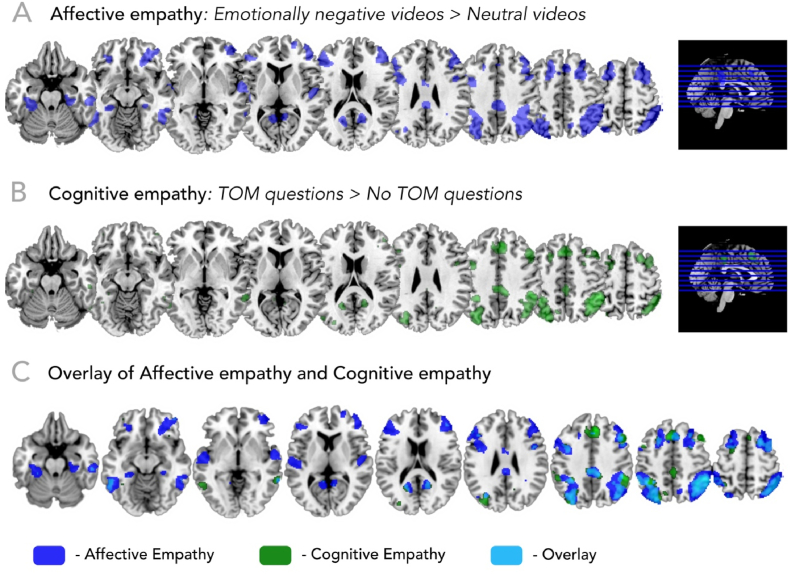
(A) Brain activation for the affective empathy network (emotionally negative > neutral videos). (B) Brain activation for the cognitive empathy network (ToM > noToM). (C) Neural overlap of affective and cognitive empathy network. Results point towards a clear network dissociation between the two routes of understanding others.

Analysing the affective empathy contrast (emotionally negative > emotionally neutral videos) yielded activation in the superior division of the lateral occipital cortex, posterior supramarginal gyrus, middle frontal and inferior temporal gyri, inferior and superior temporal gyri, posterior cingulate cortex, and the fusiform and angular gyri (Table 2; Fig. 4A). These clusters largely overlap with several data-driven models published before [13,48].

**Table 2:** Activation peaks for affective empathy.

	H	MNI coordinates			T	k
		x	y	z		
***Emotionally negative > neutral videos***						
Posterior cingulate gyrus	L	0	−32	34	18.3	523
Middle frontal gyrus	R	48	40	22	16.9	1708
	L	−46	38	20	12.5	1402
Superior parietal lobule	R	34	−56	46	16.3	3097
Angular gyrus	R	32	−64	48	14.7	
	L	−30	−64	42	15.8	2250
Superior frontal gyrus	R	24	18	58	16.1	1258
	L	−20	12	52	13.4	746
Middle frontal gyrus	R	28	28	48	8.8	
Supramarginal gyrus	L	−28	−76	38	12.2	
Inferior temporal gyrus	L	−54	−48	−12	15.7	520
	R	56	−48	−10	14.5	539
Fusiform gyrus	L	−28	−36	−20	14.1	350
	R	30	−28	−22	7.68	
Planum polare	L	−50	−12	0	12.2	537
Precuneus	L	−10	−54	12	13.1	324
	R	10	−50	12	11	254
Precuneus/Superior parietal lobule	R	12	−62	52	6.8	64
Superior temporal gyrus	R	62	−2	−6	10.4	340
Supplementary motor cortex	R	4	24	46	10.2	163
Anterior orbital gyrus	L	−26	36	−14	10.1	153
Parahippocampal gyrus	R	26	−32	−16	9.1	174
Precentral gyrus	R	46	8	30	7.9	126
Middle cingulate gyrus	L	−4	2	30	7.3	52
	R	6	−2	30	6.9	
						
***Emotionally neutral > negative videos***						
Superior frontal gyrus	L	−4	48	30	26.8	5610
Precuneus	L	0	−56	36	21.51	2143
Posterior cingulate gyrus	R	8	−48	28	14.9	
Angular gyrus	L	−48	−58	10	20.5	16571
	R	52	−50	24	18.2	
Supramarginal gyrus	L	−56	−54	28	18.2	
Middle cingulate gyrus	L	−2	−16	40	12.9	433
Middle frontal cortex	R	2	54	−16	12.1	334
	L	−34	24	42	9.3	472
Anterior cingulate gyrus	L	−2	24	22	9.8	132
Lingual gyrus	L	−4	−64	−2	9.4	913
Fusiform gyrus	R	34	−52	−20	8.03	
Precentral gyrus	R	44	−2	48	8.5	485
Occipital pole	L	−16	−98	24	7.3	43
Middle occipital gyrus	L	−40	−88	10	7.1	37
Inferior occipital gyrus	R	44	−82	−6	6.5	36
Supplementary motor cortex	R	4	−2	64	6.3	39

#### 3.3.3 Cognitive empathy

The analysis of the cognitive empathy contrast (ToM > noToM questions) yielded activation in the bilateral TPJ, middle frontal gyrus, MPFC, superior frontal gyrus, precuneus/posterior cingulate cortex, middle and inferior temporal gyri, temporal pole (Table 3; Fig. 4B). Similar to the affective empathy networks, these clusters partly overlap with a meta-analysis of ToM studies [13].

**Table 3:** Activation peaks for cognitive empathy.

	H	MNI coordinates			T	k
		x	y	z		
***ToM > noToM questions***						
Superior frontal gyrus	R	6	34	38	11.9	1973
	R	28	58	24	6.6	31
	L	−22	6	66	8.16	489
Supramarginal gyrus	R	52	−44	42	11.9	2070
Angular gyrus/superior temporal lobule	L	−28	−74	46	10.9	2362
Middle temporal gyrus	L	−62	−50	−8	10.2	338
	R	60	−44	4	8.23	135
	R	64	−28	−16	7.63	97
Posterior cingulate gyrus	L	0	−32	40	9.85	434
Fusiform gyrus	L	−28	−34	−18	8.14	47
Middle frontal gyrus	R	48	40	22	7.9	81
	L	−38	6	38	7.2	169
	L	−48	28	24	6.8	63
Precuneus	R	16	−52	20	7.1	120
	L	−18	−56	18	6.8	112
Precentral gyrus	L	−44	4	24	6.7	31
						
***noToM > ToM questions***						
Superior temporal gyrus	R	62	−2	−6	21.3	16515
Superior frontal gyrus	L	−8	58	24	11.9	2161
	L	−10	24	62	7.9	74
Precuneus	L	0	−56	36	10.9	548
Medial frontal cortex	R	0	54	−14	10.2	499
Supplementary motor cortex	L	−4	−2	64	8.5	257
Occipital pole	L	−16	−96	24	8.25	114
Precentral gyrus	L	−42	−6	62	7.6	75
Superior parietal lobule	R	22	−48	78	6.9	44
Postcentral gyrus	L	−24	−38	68	6.2	49

## 4 Discussion

This study investigated the effect of 100 mg transdermal testosterone administration on cognitive and affective empathy. As predicted, testosterone administration significantly increased serum testosterone concentrations relative to baseline and placebo as soon as 1.5 h following treatment. Independent of testosterone administration, the task consistently induced affective and cognitive empathy behaviourally and at the neural level as highlighted by changes in subjective valence ratings and functional activity of brain areas canonically associated with affective and cognitive empathy. Behaviourally and independent of testosterone administration, participants reported higher affective responses and more empathic concern in response to negative relative to emotionally neutral videos as well as to negative ToM compared to noToM videos. Unlike our predictions, however, testosterone administration did not alter the functional activity of brain networks associated with affective and cognitive empathy. Although testosterone administration did not affect the brain networks supporting cognitive and affective empathy processes, testosterone administration influenced the empathic concern and led to increased confidence in own responses regardless of response accuracy.

The task induced both cognitive and affective empathy as indexed by changes in subjective valence ratings for negative affect and empathic concern and also by the functional activation of the canonical empathy networks. Functional activity associated with affective empathy was observed in the ACC, medial prefrontal cortex, inferior frontal gyrus, and the dorsal TPJ. These structures are part of a core network activated in response to witnessing sufferance in others which is consistent with the results of empathy for pain meta-analyses [[13], [23], [30], [51]]. Several studies showed that these areas are activated not only during observing someone else's emotions but also when participants experience emotions immersively [45]. This suggests that affective empathy is supported by shared network activity that may be domain general in social cognition [1,2]. Specifically, perceiving emotional expression automatically captures attention and activates corresponding somatosensory and motor representations that further facilitate emotion decoding. For cognitive empathy, we found brain activation in the bilateral ventral TPJ, temporal pole, precuneus, STS, and the medial prefrontal cortex. Our results closely match the networks highlighted in previous validation studies [31,33]. Since participants were not asked to reflect upon the actors' mental states, our results may reflect spontaneous mentalizing. While the neural networks of emotional and cognitive empathy can be differentiated, how the networks (causally) influence each other remains open for investigation.

We found no testosterone effect on the functional activity of affective and cognitive empathy networks. Although a couple of studies showed a detrimental effect of testosterone on empathy levels [14,56], these studies either assessed bioavailable testosterone levels using proxies or exogenously manipulated testosterone levels in women. The current results, however, align to findings from a recent large-cohort behavioural study showing no effect of testosterone administration on cognitive empathy across two samples of 243 and 400 healthy men [38]. Nadler and colleagues argue that the heterogeneous results concerning the indirect link between testosterone and one's ability to understand others likely reflect caveats and limitations with published studies (incl. statistical power concerns, lack of replicability across experiments, or weak moderating effects of proxies measuring prenatal testosterone exposure assessed with the digit ratio). While our neuroimaging results support Nadler et al.‘s conclusion, our findings should be interpreted with caution considering the sample size. Although both tasks (i.e., EmpaToM and the RMET reported by Nadler and colleagues) investigate the affective empathy component, evidence suggests that the tasks investigate different neural processes that are not easily comparable. Thus, the results need to be interpreted with caution since the dependent variables are not the same (cf. [47,48]. Next to this study's inherent sample size limitation, a different reason explaining these results might be that the chosen testosterone dosage was too low to elicit functional changes within the first 1.5 h following treatment administration. However, empirical support for this argument is sparce and inconsistent given previous findings showing that even smaller dosages (50 mg) can influence functional brain activity [60,61]. Alternatively, it may be that increased testosterone blood serum concentrations may not be directly mirrored by functional changes at brain level within our chosen 1.5 h time-window.

The task is relatively difficult to perform as indexed by high error rates. Compared to placebo, testosterone administration reduced error rates (37.6% vs 44%). This suggests a role of testosterone in stimulus encoding [29] through its effect on the dopaminergic system [28]. Testosterone alters neural excitation through membrane receptors over the course of several hours [24]. As dopamine transmission is testosterone-sensitive, testosterone may therefore act as an intrinsic dopamine agonist [36] enhancing cognitive performance. This argument, however, warrants further investigation and should therefore be considered with caution as most studies to date focus on the effects of endogenous testosterone on learning efficiency.

In exploratory analyses, we found that testosterone administration increased men's self-reported confidence in their own responses regardless of their response accuracy. Evidence shows that low prenatal testosterone likely leads to overestimating task performance in tasks requiring strategic performance, with overconfident men consistently gaining fewer earnings than men who conservatively estimated their expectations [17]. Nevertheless, the correlation between testosterone exposure and self-confidence may not necessarily highlight a causal relation. Instead, other factors could independently modulate this relationship. For instance, preliminary evidence shows that cultural differences may moderate this effect with self-construal (or the extent to which one is defined independently or interdependently of others) being one of the most important moderators [62]. Future investigations should parse out the role of self-construal in mediating the relationship between self-esteem and testosterone as the current study design does not allow it.

A point of consideration is that this study was performed with a healthy and predominantly white male sample which is not representative of the general population. Likewise, these findings cannot be generalized to the other sex. Moreover, to increase the ecological validity of our pharmacological manipulation, we administered transdermal testosterone using an established protocol to elevate serum testosterone slightly over the (upper) normal male physiological range. Last, there is evidence that prenatal testosterone plays a role albeit weak in the activational effects of testosterone for higher-order social cognition [54]. As such, future investigations parsing out the effects of androgens on cognitive and affective empathy should control, amongst others, for prenatal androgen exposure. As this is the first study to examine the effects of testosterone administration on simultaneously measured cognitive and affective empathy, further investigations with larger samples are required to resolve the inconsistencies in the literature, replicate, and expand upon the current findings.

There is still ongoing debate regarding the extent to which or even whether testosterone administration alters social cognition. We examined the causal effect of 100 mg testosterone administration on cognitive and affective empathy and found no evidence of a testosterone administration on the canonical brain networks supporting the two routes to understanding others in healthy young men. Relative to placebo, testosterone administration decreased error rates during task performance and increased self-confidence in own responses regardless of response accuracy. Even though in this study testosterone did not alter the brain activity underlying affective and cognitive empathy, it did influence socio-cognitive processes (i.e., empathic concern, self-confidence). Although the task still pends careful scrutiny regarding its diagnostic value and utility, the paradigm allows for independent manipulation of both affective and cognitive empathy and can be used to expand our understanding of the interplay between mentalizing and affect sharing in clinical contexts. The reproducibility and variability of the current and previous findings should nevertheless be addressed in upcoming experiments.

## Author contributions

Conceptualization: AAP and KK.; methodology, analyses, and visualization: AAP and MV; writing—original draft: AAP; writing—review and editing: AAP, MV, UH, and KK. All authors have read and agreed to the published version of the manuscript.

## Funding

This research was funded by the German Research Foundation (DFG—269953372/GRK2150). AAP was supported by a fellowship from the International Research Training Group—The Neuroscience of Modulating Aggression and Impulsivity in Psychopathology (IRTG-2150) International Research Training Group—The Neuroscience of Modulating Aggression and Impulsivity in Psychopathology (IRTG-2150). The funding source had no role in the design of this study, its execution, analyses, interpretation of the data, or the decision to submit results.

## Institutional Review Board statement

The study was conducted according to the guidelines of the Declaration of Helsinki, and approved by the Institutional Review Board (or Ethics Committee) of the Medical Faculty of the RWTH Aachen University.

## Informed consent statement

Written informed consent was obtained from all subjects involved in the study.

## Data availability statement

Data may be made available upon request to the corresponding author.

## Conflicts of interest

The authors declare no conflict of interest.

## Declaration of interest

None.
